# Genomics-Assisted Breeding for Quantitative Disease Resistances in Small-Grain Cereals and Maize

**DOI:** 10.3390/ijms21249717

**Published:** 2020-12-19

**Authors:** Thomas Miedaner, Ana Luisa Galiano-Carneiro Boeven, David Sewodor Gaikpa, Maria Belén Kistner, Cathérine Pauline Grote

**Affiliations:** 1 State Plant Breeding Institute, University of Hohenheim, Fruwirthstr. 21, 70599 Stuttgart, Germany; ana.galiano@kws.com (A.L.G.-C.B.); david.gaikpa@uni-hohenheim.de (D.S.G.); kistner@agro.uba.ar (M.B.K.); catherine.pauline.herter@gmail.com (C.P.G.); 2 Kleinwanzlebener Saatzucht (KWS) SAAT SE & Co. KGaA, 37574 Einbeck, Germany; 3 Estación Experimental Pergamino, Instituto Nacional de Tecnología Agropecuaria (INTA), CC31, B2700WAA Pergamino, Argentina; 4 Consejo Nacional de Investigaciones Científicas y Técnicas (CONICET), Godoy Cruz 2290, C1425FQB Buenos Aires, Argentina

**Keywords:** resistance breeding, small-grain cereals/*Fusarium* head blight, wheat/*Septoria tritici* blotch, wheat/*Septoria nodorum* blotch, maize/*Gibberella* and *Fusarium* ear rot, maize/Northern corn leaf blight, multi-disease resistance (MDR), genetic resources

## Abstract

Generating genomics-driven knowledge opens a way to accelerate the resistance breeding process by family or population mapping and genomic selection. Important prerequisites are large populations that are genomically analyzed by medium- to high-density marker arrays and extensive phenotyping across locations and years of the same populations. The latter is important to train a genomic model that is used to predict genomic estimated breeding values of phenotypically untested genotypes. After reviewing the specific features of quantitative resistances and the basic genomic techniques, the possibilities for genomics-assisted breeding are evaluated for six pathosystems with hemi-biotrophic fungi: Small-grain cereals/*Fusarium* head blight (FHB), wheat/*Septoria tritici* blotch (STB) and *Septoria nodorum* blotch (SNB), maize/*Gibberella* ear rot (GER) and *Fusarium* ear rot (FER), maize/Northern corn leaf blight (NCLB). Typically, all quantitative disease resistances are caused by hundreds of QTL scattered across the whole genome, but often available in hotspots as exemplified for NCLB resistance in maize. Because all crops are suffering from many diseases, multi-disease resistance (MDR) is an attractive aim that can be selected by specific MDR QTL. Finally, the integration of genomic data in the breeding process for introgression of genetic resources and for the improvement within elite materials is discussed.

## 1. Introduction

Plant breeding aims to develop new cultivars with superior performance in terms of grain yield, disease resistance and grain quality. Per crop, about 10 to 30 individual traits have to be considered in a multi-step procedure where different traits are selected in different generations, starting with one-row plots in early generations and ending with large-drilled plots (5–10 m^2^) in late generations. Selection starts with a plethora of 100,000s of genotypes and should end with 1–5 successful candidates per year. This procedure takes 6–12 years depending on the crop and is therefore tedious and capital-intensive. Therefore, each possibility to speed up this process and to get a more targeted outcome would be highly appreciated. Besides gene transfer and genome editing, genomics could play a prominent role in future plant breeding processes. In contrast to genetics, genomics aims at the collective characterization of all genes/genomic segments and their interaction that are responsible for a quantitative disease resistance (QDR) phenotype. This became possible by developing high-density marker chips based on full-genome sequencing that has made tremendous progress in the last decade and is now available for most crops at reasonable cost. 

Disease resistance has been dichotomically classified in qualitative and quantitative resistances. Qualitative resistance is based on individual resistance (*R*) genes mainly encoding *R* proteins that interact with pathogen-specific effectors triggering a complete defensive response called effector-triggered immunity (ETI), while the molecular basis of QDR is poorly understood. Hypotheses include regulation of morphology and developmental traits, basal defense, production of anti-fungal compounds, defense signal transduction, weak ETI triggered by ‘‘defeated’’ *R* proteins, or unidentified mechanisms [1]. The evidence suggests that probably all these mechanisms can lead to QDR, but also that the division between qualitative and qualitative resistance seems to be not so contrasting as was proposed historically.

In this review, we will concentrate on QDR of the four small-grain winter crops rye (*Secale cereale* L.), triticale (×*Triticosecale* Wittm.), durum wheat (*Triticum turgidum* ssp. *durum*), bread wheat (*T. aestivum* ssp. *aestivum*), and of maize (*Zea mays* L.). These crops provide more than half of the global caloric intake [2]. Caused by the world-wide growth of human population, the consumption of cereals between 2019 and 2028 is expected to increase at 1.2% per year, with increasing demand in Asia and Africa [3]. The production of cereals also provides employment for millions of people throughout the world. The most important factors restricting further yield improvements are abiotic stress factors and pests with the latter causing a yield loss of approximately 30–40% on a world-wide basis with a large range from 5–90% depending on the disease and the region [4,5]. Without the application of chemical plant protection, the worldwide loss could rise, on average, to 50–75% depending on the crop [4]. From the side of diseases, we will concentrate on six hemi-biotrophic fungal diseases that are the most pertinent threat in temperate regions, among them are some of the most important plant pathogens worldwide: Small-grain cereals/*Fusarium* head blight (FHB), wheat/*Septoria tritici* blotch (STB), and wheat/*Septoria nodorum* blotch (SNB), maize/*Gibberella* ear rot (GER) and *Fusarium* ear rot (FER), maize/Northern corn leaf blight (NCLB). The papers reviewed here should provide a wider view on QDR by analyzing large numbers of genotypes with a full-genome coverage concentrating on the last five years where the most dramatic progress was made.

## 2. Genetics of Quantitative Disease Resistance (QDR)

Quantitative disease resistances (QDR) are called quantitative because they are distributed in segregating generations in a quantitative manner [6,7]. They are typically caused by many genes with moderate to small effects that are highly affected by environment, plant organ, and plant developmental stage. Since the advent of genomics, we know that QDR to most pathogens is caused by hundreds of quantitative trait loci (QTLs) that are distributed across the whole genome. QDR results from a restriction in the growth or development of the pathogen imposed by the host, and theoretically it should be measured by determining the amount of pathogen in the host tissues [8]. In practice, this is not feasible for large populations and typically, QDR is determined in multi-environmental field trials by visual symptom rating with the inclusion of standard varieties of known resistance level. Each genotype that is performing significantly better than a susceptible standard is called ‘quantitatively resistant’. The degree of QDR can vary from very small to nearly full resistance. Therefore, quantitative scales of disease assessment, which are good estimators of the pathogen development, are inevitable. This could be a simple 1 to 9 scale as preferred by plant breeders to a 0–100% scale that allows a finer resolution. Depending on the growth stage, the same plant genotype can have different QDR to the same pathogen species. This is notorious for *Fusarium* diseases that can affect practically all cereal stages and organs, but the correlations among the respective QDR are always poor illustrating that different sets of genes are responsible where most (but not all) are stage specific. Most important is the adult-plant resistance. Typically, QTLs are providing a non-race specific resistance that is effective against all genotypes of a pathogen or even against different pathogens (multi-disease resistance, MDR). The molecular basis of QDR is poorly understood, mainly because it is a multi-facetted defense system that involves hundreds of (unspecific) cellular processes, like cell-wall thickening (lignin, callose, glycoproteins), phytoalexins, reactive oxygen species (ROS), pathogenesis-related (PR) proteins (chitinases, glucanases), inhibitors of fungal enzymes, detoxification of mycotoxins, lack of/different toxin receptors [9]. Although some of these pathways are also used by monogenic, qualitative resistance genes, QDR typically does not comply a complete resistance and shows no symptoms of hypersensitivity. This led to the hypothesis that QDR genes are defeated qualitative genes that have retained some resistance activity [10]. Although this might apply for some specific examples, it cannot explain the plethora of hundreds of QDR genes detected per pathosystem [11]. Because of the complex inheritance and their non-race specifity, QDR is thought to be of higher durability than qualitative disease resistances that are notorious for being overcome by single mutations in the pathogen population. Although it is impossible to prove durability experimentally before the release of cultivars, there are examples where QDR holds for many years [12].

QDR must be strictly separated from a few so-called pleiotropic APR genes that provide in wheat a non-hypersensitive adult-plant resistance against all races of multiple pathogens, because they are clearly monogenically inherited as shown by recent gene cloning, like *Lr34/Yr18/Sr57/Pm38* [13] or *Lr67/Yr46/Sr55/Pm39* [14]. Typically, they are not providing a complete resistance, but display a partially resistant phenotype, thus resembling QDR.

## 3. Basic Techniques for Genomics-Assisted Breeding

**Figure 1:**
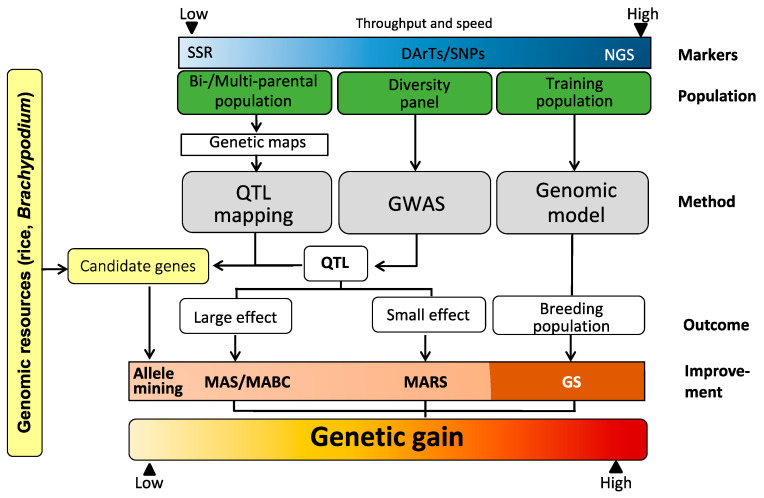
Techniques for genomics-assisted breeding (based on an idea of Bohra et al. [15], adapted). SSR = Single sequence repeat, DArT = Diversity Array technique, SNP = Single nucleotide polymorphism, NGS = Next-generation sequencing, QTL = quantitative trait locus, GWAS = genome-wide association studies, MAS = Marker-assisted selection, MABC = Marker-assisted backcrossing, MARS = Marker-assisted recurrent selection, GS = Genomic selection.

Genomics include several techniques that are all based on marker assays of varying density or sequence data but the methods are highly different from their genetic requirements and biometrical foundations (Figure 1). It all started in the 1980s with QTL mapping by the analysis of bi-parental populations with a density of about 100 markers. Starting with a F_1_ plant, either higher selfing generations or double-haploid (DH) libraries are produced without any selection to map segregating QTLs. 

The precision of QTL mapping is mainly governed by the heritability of the trait, the population size, and the marker density. QTL mapping is stroke by a high bias that (1) regularly overestimates the QTL effect by about 50% and (2) does not allow to pinpoint the locus to a small chromosomal segment [16]. A more recent advantage is the use of multi-parental populations that overcome the problem that only two parental alleles can be analyzed in a specific background. From the definition, at least one parent should connect the crosses [17,18]. This is also more convenient for breeding purposes where many crosses are made, but usually only with small population sizes.

Nowadays the problem of marker density is solved by low-cost medium- to high-density marker assays for all cereals providing 5000 (rye) to 600,000 (maize, rye) markers in one run. This allowed in 2005 the first genome-wide association study (GWAS) for disease resistances in *Arabidopsis* [19] and later in all important crops. GWAS is based on diversity panels and takes advantage from historic recombination events. Thus, much more accessions are screened with the possibility to identify new alleles not represented in parents of bi-parental mapping populations [20]. The outcome of a GWAS is mainly dependent on population size, but also on the crop- and genome-specific linkage disequilibrium (LD) between marker and phenotype. As a rule of thumb, the marker density must be higher the faster the LD decays. Consequently, a fast decay of LD allows a much more precise localization of the QTLs than QTL mapping when marker density is sufficiently high. QTLs with non-additive genetic effects or QTLs with rare alleles could, however, not be detected by routine GWAS.

QTL mapping and GWAS result in the detection of QTLs that can be used either directly for marker-assisted selection (MAS) or marker-assisted backcrossing (MABC), when the effects are high enough and reproducible in unrelated populations or for marker-assisted recurrent selection (MARS) when the effects are only small. The latter has been shown to be successful for resistance to Fusarium crown rot caused by *Fusarium pseudograminearum* that has a quantitative, complex inheritance [21]. 

Due to the availability of large genomic resources for grass model plants, for example rice, *Brachypodium*, candidate genes can be predicted from QTLs and further functionally analyzed. Possible candidate genes can be deduced by searching sequence data bases when the confidence intervals of the QTLs are small enough, i.e., the study should be done with large populations, high marker density and a high precision of phenotyping. With cloned genes, allele mining in large breeding populations or gene banks is possible to detect alternative alleles that might provide a lower host specificity or higher expression level. Allele mining is also useful to enhance allele diversity and to extend possibly the durability of these genes. For QDR, however, the postulation of candidate genes is still highly speculative in most cases.

The landmark paper of Meuwissen et al. [22] opened the avenue for genomic selection (GS) in animal breeding, that was later adjusted also for plant breeding purposes [23]. Based on high-density marker assays, the whole genome is scanned for effects on the investigated traits and further used to estimate the genomic breeding value of individuals to be selected. Once the marker effects are estimated in a large training set that is used to train GS models (= training set), non-tested genotypes (= validation set) can be predicted and selected based on of their genome composition. This strategy reduces large-scale phenotyping and enhances selection gains [24,25] and is especially valuable when the trait is mainly controlled by a multitude of additive alleles with small effects [26]. A major requirement for GS is a high genetic relationship between training and validation population that makes it necessary to repeat the model training after each introgression of non-related material. In many scientific papers, no real selection occurs, but predictions of the potential outcome are made (= genomic prediction, GP). Factors, limitations and prospects of GS for quantitative traits have been extensively reviewed (Table 1) [24,25,26,27]. The main criterion for the quality of a prediction is the prediction ability, i.e., the correlation between the genomically predicted and the phenotypically estimated trait values. When the prediction ability is divided by the square root of the heritability, this is called prediction accuracy.

All genomic methods require extensive phenotyping with high precision because this is the basis for estimating exact marker effects. This affords the analysis of large populations over several locations and years that should reflect the future target environments. This is also the only way for identifying environmentally stable QTLs that are the core element in applied breeding programs [28]. Phenotypic evaluations for QDR are usually assessed in the field in adult-plant stage and should include artificial inoculation to ensure high disease pressure and uniform disease distribution in the experiment. Inoculation methods should be feasible for analyzing large populations and can only be dispensed when the environments regularly allow a high disease severity. High-throughput phenotyping, for example by image analysis, offers a future perspective to improve trait assessment in quantitative resistances and could make it faster and more accurate [29].

A problem of most genomic studies is an overestimation of prediction accuracy due to various reasons (Table 1). Often, the cross-validation of genotypes is evaluated within the same environments (locations, years). Or the detected QTLs were not validated either in independent genetic materials or at least in an independent fraction of the genetic materials where they have been detected, e.g., in backcross generations or by independently resampling the original populations [16]. Overestimation is also given when a high degree of relatedness between validation and training sets occurs. In many papers 80% of a given data set is taken as training set and used to fit a GS model to predict the remaining 20% of the same data set as validation set. This sort of cross-validation is repeated several hundred times with randomly varying combinations of training and validation sets. However, this procedure does not reflect real breeding programs where a lot of new crosses of mostly small size are handled every year [28,30]. Prediction accuracies for FHB resistance in wheat, for example, dropped from a range of 0.34 to 0.63 for predictions within small bi-parental families to values from −0.31 to 0.53 for predictions across bi-parental families [31]. However, in practical breeding, individuals of the training and validation sets are mostly evaluated in different trials or environments, and very often disease symptoms are scored by different people. In this case, prediction accuracies may be lower than what has been reported in literature. Efforts must be made to optimize GS in applied breeding by constantly updating the training set when new phenotypes and marker data are available [32]. A comprehensive discussion on the different factors affecting prediction accuracy is available [33]. Thus, verification of QTLs is essential before integrating them into practical breeding programs. Techniques like Kompetitive Allele Specific PCR (KASP) or Real-Time Quantitative Reverse Transcription PCR (qRT-PCR) are available to validate the major effect SNPs before they are incorporated into breeding populations.

## 4. Advantages and Challenges in Genomics of Quantitative Pathosystems

### 4.1. Fusarium Head Blight in Small-Grain Cereals

*Fusarium* head blight (FHB) is caused by *Fusarium graminearum* as the most frequently occurring fungus worldwide, but in Europe *F. culmorum*, *F. avenaceum*, *F. poae* and others can also cause similar symptoms. The disease not only results in yield and quality reduction, but also in contamination of the harvest with mycotoxins that are harmful to humans and livestock, especially swine. FHB affects all small-grain cereals with the most susceptible being durum and bread wheat, followed by triticale and rye [34]. Also, barley and oats can be infected, however, the symptoms are not so easy to follow due to the different architecture of the head or panicle. Studies with artificial infection showed high positive genotypic correlations between FHB severity and *Fusarium* mycotoxin contaminations in maize [35], wheat and rye, but not in triticale [36]. The genetic architecture of FHB resistance is complex, affected by multiple loci, the environment and strong genotype × environment interactions (G × E) [36,37]. This limits the success from conventional breeding. 

Wheat resistance to FHB is one of the most analyzed pathosystems worldwide. In bread and durum wheat, >550 QTLs have been described in the literature [38] that could be attributed to 65 so-called meta-QTL, i.e., QTL that were detected in several studies at the same locus (so called “hot spots”). Only one meta-QTL on chromosome 3B was so refined that it could be used for detecting candidate genes. Ten candidate genes for FHB resistance were found that were differentially expressed in a resistant cultivar [38]. Two genes were encoding proteins already known to be important for resistance (glycosyltransferase, Cytochrome P450), three were newly detected without knowing their role and the other five encoded uncharacterized proteins. This clearly illustrates the potential, but also the challenges for gene identification by genomic means. In the meantime, seven prominent QTLs have been fine mapped (*Fhb1-Fhb7*) and *Fhb1* was the first gene of this complex to be cloned [39]. Most of these QTLs/genes were derived from FHB resistant Chinese wheat, esp. Sumai-3 and Wangshuibai and were used worldwide for MAS. One of these QTL, however, does not suffice for a high resistance level [40]. Four QTLs (*Fhb1, Fhb2, Fhb4, Fhb5*) from Wangshuibai were introgressed by MAS in 40 elite Chinese cultivars resulting in lines with a high resistance comparable to the donor [39]. In adapted European wheat sources, many low- to medium-effect QTLs were detected [37,38] that can also provide a high resistance level, when they are accumulated by recurrent selection procedures. They are, however, not useful for MAS because they are often population-dependent, in their majority not validated for their effects and the accumulation of several resistance FHB QTLs will lead to a fixation of large portions of the genome, thus decreasing the chance for selecting other traits [41].

For the other small-grain cereals, much less work has been done. Some QTL studies and GWAS were done in durum wheat due to its extreme susceptibility to FHB [42,43,44,45], but for triticale only a few studies [46,47,48] and for rye only one study is available [49]. In this first paper on rye, a single-locus GWAS method detected 15 QTLs among nearly 500 partially inbred lines distributed across all chromosomes except chromosome 7. These QTLs collectively explained about 74% of the genotypic variance [49]. Similar results have been found in the other small-grain cereals. 

Caused by the quantitative nature of FHB resistance and the underlying small- to medium-effect QTLs in adapted materials, genomic selection (GS)/genomic prediction (GP) should facilitate the application of genomics in improvement. A larger proportion of genetic variation may be captured by GS than by MAS. In several recent studies, GS was compared to the traditional MAS using only QTLs with marker effects of > 5% of genotypic variation. The GP approach outperformed MAS in most cases as shown by studies in triticale, rye, and bread wheat [47,49,50,51]. 

The high prediction accuracies achieved in many studies are again caused by a large average kinship between training and validation population [52,53,54,55]. It must be admitted that prediction accuracy dropped dramatically when extending the GS models to less or even unrelated materials [31,55]. When analyzing genomic estimated breeding values (GEBVs) among families in wheat, highest accuracies were achieved by predicting from one half-sib family to another, while accuracies were lowest between unrelated families and even got negative in some cases [31].

In a durum wheat study, the difference between MAS and GP was much smaller, mainly because the variation within the population was rather small and already exhausted by 7 QTLs [42]. The authors concluded that FHB resistance might be better improved by classical high- throughput recurrent phenotypic selection in durum wheat. Similarly, a comparison between phenotypic and genomic selection revealed a superior prediction ability of the phenotypic selection [43]. However, higher selection responses were found in a simulation study by using GEBVs for early generation selection, a stage where phenotypic selection is rather unreliable caused by low single-plant heritability in this pathosystem. This would also accelerate population improvement much more than it is possible with classical breeding strategies [43].

Prediction accuracies can be enlarged when including the most prominent QTLs in the GS model as fixed effects (weighted GS). Especially when only a few QTLs with small to moderate cumulative effects have been detected, the weighted GP approach results in higher predictability [31,47,56,57,58]. This is also of advantage, when some high-effect QTLs/genes have a correlated effect on FHB resistance, like the semi-dwarfing gene *Rht-D1* in wheat [59]. Then it is advantageous to combine data from a GWAS into the genomic selection model for increasing the prediction accuracy when major QTLs/genes are present [58]. 

While most studies are doing GP, a recently published study was the first to report realized gains from genomic selection for FHB and STB resistances [60]. A population of 1120 winter wheat lines was used as training set to establish the genomic model for calculating GEBVs of 2500 lines that were only genomically analyzed. As an outcome, a genomic selection advantage of 10.6 percentage points for FHB resistance was achieved compared to the randomly chosen subpopulation. 

When GS is used in practical breeding programs to select for grain yield in early generations, the prediction of QDR, like FHB resistance, is of no further costs because the marker data are available already and the phenotypic data must be collected anyway.

### 4.2. The Septorias in Wheat

Two *Septoria* diseases are prevailing in wheat: *Septoria tritici* blotch (STB), caused by *Zymoseptoria tritici* (teleomorph *Mycosphaerella graminicola*) and restricted to leaf symptoms, and *Septoria nodorum* blotch (SNB) caused by *Parastagonospora nodorum* (teleomorph *Phaeosphaeria nodorum*) and causing leaf and glume symptoms. Both pathogens are hemi-biotrophic and show a quantitative inheritance although several isolate-specific genes with gene-for-gene interaction are known for *Zymoseptoria* (*Stb 1–18*) and *Parastagonospora* (*SnTox1–7*). The latter are necrotrophic effector proteins that induce necrosis in wheat when confronted with specific wheat sensitivity genes (*Snn*) [61].

A GWAS of 225 wheat cultivars revealed that 11 of the 21 wheat chromosomes were associated with for STB resistance explaining, however, only 38% of phenotypic variation [62]. This implies a similar quantitative inheritance like FHB resistance. Further, the genetic architecture of STB resistance was analyzed by GWAS based on a mapping population of 1055 European wheat hybrids [63]. The cross-validation study confirmed that the genetic architecture underlying STB resistance in this population was complex with an absence of large-effect QTLs. Also, individual isolate-specific resistance genes have not been detected in this population, because the most common resistance genes in European wheat (*Stb6, Stb15*) have already been overcome by the European *Z. tritici* populations and are, thus, not effective anymore [57]. *Stb* genes from more exotic origin, like as *Stb1* originating from a Bulgarian landrace, or *Stb18* have not yet been introgressed into European elite wheat lines. 

The accuracy to predict STB resistance revealed only 0.3 when using a validation set mostly unrelated to the training set [63]. These results are in accordance with a later study of 1604 European wheat hybrids comparing GS for STB and FHB resistances [50]. The GWAS again revealed the absence of large-effect QTLs for both resistances. Cross-validated prediction accuracies of disease severity among unrelated hybrids amounted to 0.58 for FHB and for 0.23 for STB resistances. Among closely related hybrids, prediction accuracy increased substantially, but was still lower for STB resistance. Obviously, the quantitative STB resistance is inherited even more complex than FHB resistance [50]. Accordingly, prediction accuracies of 0.45 and 0.43, respectively, were reported when analyzing about 300 lines from different winter wheat panels [64,65]. Prediction accuracy for STB resistance in seedling stage could be improved from 0.47 to 0.62 when all non-redundant GWAS markers were used as fixed effects [57]. 

This highly complex genetic architecture of the wheat host might counterbalance the huge genetic variation of the pathogen. This and the absence of monogenic resistance governed by *Stb* genes suggests that resistance in European material is durable, because of the inheritance by a high number of genes thus reducing the risk of the resistance to be overcome by race-specific isolates [63]. Given the low accuracies of GS from two studies with vast wheat populations (> 1000 entries, [50,63]), phenotypic selection of quantitative STB resistance might be more encouraging than GS approaches.

The genetic control of resistance to SNB is also very complex, consisting of many loci with additively inherited minor effects and prone to high genotype × environment interactions resulting in only low to moderate correlations among environments [20,66]. Therefore, only a few QTLs have been detected across environments (locations, years). For flag leaf resistance, QTLs on chromosomes 1B, 2A, 2D, and 5B and for glume resistance on chromosomes 2D and 4B were detected in successive years [67]. A newer study with 232 genotypes of global origin detected 20 QTLs on nine chromosomes with most QTLs detected only in one environment [20]. Only four QTLs provided resistance to several isolates in specific environments. Accordingly, in a panel of Nordic spring wheats most of the QTLs were detected in only one field environment and only two QTL on chromosome 2A and 2D were found in all environments [66,68]. A QTL on chromosome 2A additionally provided resistance to both leaf and glume blotch [68]. In addition, eight QTLs were identified in seedling stage, but only two of them on chromosomes 4B and 7A were also significant in adult-plant stage. Considering the different developmental stages (seedling, adult plant), the different plant organs (flag leaf, glume), and the high interactions with isolates and environments, wheat resistance to SNB is extremely variable and seems to be even more complex than for STB resistance explaining also the slow genetic progress by traditional breeding [20].

### 4.3. Gibberella and Fusarium Ear Rots in Maize

Ear rots are major diseases in all maize-growing areas worldwide. However, the fungal species causing the diseases are quite different. In the temperate zone of the Northern hemisphere, *F. graminearum, F. verticillioides*, and *F. temperatum*, a new species separated from *F. subglutinans*, are the main species causing ear rots [69]. The composition of species in an actual year is mainly associated with weather conditions during silking. *F. graminearum* (teleomorph *Gibberella zeae*) causing Gibberella ear rot (GER) prevails with cooler temperatures, the other species causing Fusarium ear rot (FER) (teleomorph *G. fujikuroi*) are more prone to higher temperatures. Unfortunately, all mentioned species produce chemically different mycotoxins with deoxynivalenol (DON) and zearalenone (ZON) being the most prominent for *F. graminearum* and fumonisins (FUM) the most prominent for *F. verticillioides*. All *Fusarium* species can infect via the silk channel during silking period and via wounds in the cob leading to kernel infection. With rising temperatures due to global climate change the proportions of isolated pathogens might shift to FER-causing fungi also in northwestern Europe [69] and additionally the damage by insects might increase in frequency and severity providing an entry portal for *Fusarium* spp. For these reasons, the identification of QTLs that are common among different fungal pathogens should be favored in future to establish a broader resistance. A review on genomic studies known to date can be found for GER [35] and FER [70].

For GER and FER, uniquely QDR has been identified to date. Several studies based on adapted germplasm identified many QTLs that explained together 21 to 59% of the total genotypic variance [35,55,70,71,72,73]. Overlapping QTLs between GER and DON concentration are expected as both traits were highly correlated (*r* > 0.86) [74,75]. Similarly, GER and ZON concentrations were correlated (*r* = 0.91) [76]. Indeed, two QTLs with large effects each explaining 29 to 35% of the total genotypic variance were found on bin 1.11 and 2.04, respectively, that provided resistance against GER and additionally a low contamination by DON and ZON [77]. Similarly, between FER resistance and FUM concentrations high genotypic correlations were found (*r* = 0.74–0.84) [78]. However, also specific genes might additionally play a role in GER resistance and reduced mycotoxin accumulation [35]. Nevertheless, for the large populations handled by practical breeding in early stages, a selection for low ear rot severity should suffice. The much lower number of experimental hybrids in later stages could be analyzed also for mycotoxin contents, e.g., by NIRS or immunotests [75]. 

In a first validation study for QTLs on GER resistance, six QTLs identified in a previous mapping study were introgressed into two different genetic backgrounds [79]. The validation rate was, unfortunately, low which indicates that the QTLs identified were population specific. This is further substantiated by the fact that different QTLs were validated across several bi-parental mapping populations. However, two meta-QTL analyses resulted in common QTLs localized in bins 2.08, 3.04, and 4.08, some of them were valid even for GER, FER and Aspergillus ear rot resistance together [80,81]. 

Genomic analyses of exotic germplasm may introduce new sources of resistance alleles to adapted European germplasm [35]. Tropical and subtropical maize as well as popcorn populations are possible sources of resistance alleles for ear rot for temperate maize breeding pools [82] and should be explored to achieve higher resistance levels. However, only a few studies exploiting genetic resources to increase GER resistance have been reported previously (e.g., [80,82]). By conducting multi-parent QTL mapping, one QTL on bin 1.02 was identified which was overlapping across several populations and continents (Brazil/Europe) as well as across environments within continents and across line and testcross performance with an explained genotypic variance of 10–22% depending on the situation [83]. In total, four QTLs have been found in this study within Brazilian or European environments. This low number of QTL might be caused by the high diversity of European environments in Germany, Austria, Northern France and Northern Italy or just illustrate the highly polygenic and complex genetic architecture that even could hinder to find any QTL in a GWAS [78].

Another valuable genetic resource are European flint landraces. Higher phenotypic variation and broad-sense heritabilities within landraces than among elite lines were reported for FER severity [84] by evaluating 389 DH lines from six European flint landraces and 53 elite flint lines in a GWAS. Also in a second study where a GWAS was undertaken with 500 DH lines from two flint landraces, maximum phenotypic variation was found in the Austrian landrace “Kemater Landmais gelb” (KE) and the German landrace “Petkuser Ferdinand rot” (PE) [85]. In the GWAS, however, PE showed no significant QTL, while KE revealed eight QTLs explaining together 34% of genotypic variation. Interestingly, a GP procedure revealed similar prediction accuracies for lines from both landraces implying that many small effects failed to pass the significance threshold due to limited detection power. The GP procedure weighted with the most significant QTLs was about 20% better than MAS in KE [85]. GP was also recommended for improving FER resistance by claiming the shortening of generation intervals and reducing laborious QDR evaluation in the field as main advantages [86]. 

Genetic relationship between training and validation populations plays a major role also in maize. When analyzing European maize with 130 dent lines and 114 flint lines for GER resistance and DON contents, prediction accuracies for DON content were 0.66 within the dent pool and 0.45 within the flint pool [72]. They dropped for the prediction across pools to 0.1 for the dent lines and even got negative for the flint lines. Accordingly, no common QTL was localized in the two European heterotic groups, flint and dent. A combined-pool GP had no higher accuracy than within-pool GP, regardless of the statistical model used. In accordance with this finding, using only DH lines from one landrace to predict GER resistance in the other landrace was also not promising at all [85]. In another study involving six European maize landraces, GP between pairs of DH libraries resulted in prediction accuracies of approximately zero for all landraces and six agronomic traits analyzed [87]. However, prediction accuracies improved when the TS and vs. contained lines from both landraces.

A possible solution to optimize results from genomic studies is to combine different analytical methods to overcome the inherent weaknesses of each individual method [35,81]. For example, candidate genes for GER resistance among recombinant inbred lines derived from bi-parental crosses were identified by combining QTL mapping with transcriptomic (RNA-seq) approaches [88]. Also, QTL results can be used to predict candidate genes by *in silico*-mapping. A major prerequisite, however, is that the QTL region is small enough. Therefore, after a normal QTL study, a fine mapping should be done to further restrict the QTL region. This is especially valid in cross-pollinated crops like maize because the QTL interval can be narrowed down here rather effectively. Also, association mapping provides a complementary tool for identifying candidate genes when a region was fine mapped. By using diversity panels, significant SNPs within the fine-mapping interval can be found where the underlying candidate genes might be detected [89]. The analyzed diversity panel was then used as training population for GP. Candidate genes can be further investigated by expression analyses, resequencing, and testing across different germplasm sets [90].

### 4.4. Northern Corn Leaf Blight (NCLB) in Maize

Northern corn leaf blight (NCLB) is caused by the hemi-biotrophic ascomycete *Setosphaeria turcica* (syn. *Helminthosporium turcicum*, anamorph: *Exserohilium turcicum*). In Europe, NCLB was originally restricted to the Mediterranean regions, but at the 1990s the disease crossed the Alps and appeared in southern Germany in 1995 [91]. The disease rapidly expanded and became the most important leaf disease of maize in Northwestern Europe. The fungus and the disease are also distributed in many other maize-growing areas [91,92].

Resistance to NCLB can be both qualitatively and quantitatively inherited [92]. The employment of qualitative resistance genes (mainly *Ht1, Ht2, Ht3*) already led to directional selection in the European pathogen populations and resulted in regionally different virulence patterns [93] making some of the main *Ht* genes and their combinations already ineffective. QDR, therefore, should provide a more durable resistance. In all QTL studies reported so far, many small to medium-sized QTLs have been reported distributed across all ten maize chromosomes with mainly additive gene action [92]. In several mapping studies, four to 14 QTLs have been assigned to mean disease severity. Additionally, 12 and 19 QTLs were identified for the resistance traits area under disease progress curve (AUDPC) and final disease rating, respectively, with some QTLs being assigned to two to three traits simultaneously [94]. This corresponds to high phenotypic correlations among the traits (*r* ≈ 0.8) [95].

High levels of quantitative NCLB resistance have been detected in non-adapted (sub)tropical materials [96,97,98] that might be used for widening the resistance diversity in European maize. In a multi-parental QTL mapping, 17 QTLs distributed along the ten chromosomes were identified, each QTL explaining 3.6 to 32.0% of the genotypic variance [98]. Most of the resistance alleles originated from Brazilian donors and reduced NCLB severity between 0.3 to 2.5 scores on the 1–9 severity scale. None of the known *Ht* genes have been identified in this genetic material by associated markers or known DNA sequences. Because always local pathogen populations have been used comprising a wide range of virulences [98] the described resistances should be quantitatively inherited. This is important, because otherwise the occurrence of effective monogenic resistances would mask small, quantitative effects. Two QTLs on bins 7.03 and 9.04 were identified in Brazil and Europe as well although ecosystems were highly distinct, illustrating another form of QTL stability. As in the other crops, prediction accuracies for NCLB dropped, on average, from 0.55 for within-family prediction to 0.20 when totally unrelated materials were predicted [98]. Because of the long-standing epidemic infections of subtropical maize with NCLB there should be more resistance sources to detect after this pivotal first study.

**Figure 2:**
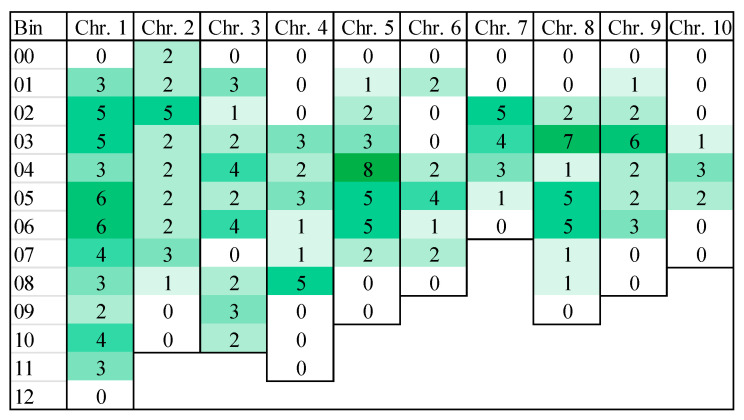
Number of QTLs for resistance to NCLB according to the literature; the intensity of color represents the number of QTLs found in the same bin (for details please refer to Supplementary Materials).

At least 197 QTLs were reported for NCLB resistance to date when combining 27 publications. Only QTLs resulting from the analysis of at least two environments and a minimal population size of 100 genotypes were considered (see Supplementary Materials). Basically, all chromosomes were involved in resistance QTLs. Localizing these QTLs in the maize genome clearly shows that there are hotspots for this resistance with up to 7 QTLs detected in the same bin (Figure 2).

In such studies, also agronomic traits should be considered that might interact with QDR, especially plant height and genes for plant development. Several QTLs controlling maturity and the gene *vgt1* (flowering time) were also mapped on bin 8.05 [98,99,100]. The presence of QTLs for resistance and maturity on the same bin could reflect resistance in late maturity genotypes because of the preference of *S. turcicum* for senescent tissue. 

These hotspots could correspond to a common gene or to multiple clustered genes [29,101] and emerge as promising regions to explore the underlying resistance mechanisms with a higher resolution [90,102]. For example, on bin 1.02, the gene *ZmREM6.3* encodes a REM protein. These proteins regulate the size exclusion limit of plasmodesmata and could restrict the movement of the pathogen in the host [102]. Some of the hotspots might be caused by the independent detection of the same QTL in different populations. This can be interpreted as a sort of independent validation that could be valuable for introgression breeding.

## 5. Detection of Multi-Disease Resistance (MDR)

All crops are affected by more than one disease at the same time. Thus, loci conferring multi-disease resistance (MDR) should be under a strong selection pressure and are a highly valuable goal for plant breeding. MDR, also called broad-spectrum resistance, was defined as the resistance of a host either to many races of one pathogen (= non-race specific resistance) or resistance to more than one pathogen species [103]. In this review we will concentrate on the latter definition. MDR might be genetically controlled by pleiotropy, pyramiding of several unlinked genes/QTLs conferring resistance to single diseases, or the presence of clusters of resistance genes in the genome [103]. A fourth, special cause could be the introgression of alien chromosomes that show a reduction or even absence of recombination, like in wheat translocation lines with the rye chromosome 1RS segment, where several rust and mildew resistances are located [104]. For the other mentioned cases, also practical examples are known from wheat. Pleiotropic broad-spectrum resistance genes, like *Lr34*/*Yr18*/*Sr57*/*Pm38*, *Lr46*/*Yr29*/*Sr58*/*Pm39*, *Lr67/Yr46* and *Sr2*/*Yr30*, are widely exploited in international breeding [105]. They confer a moderate level of MDR to leaf rust (*Lr*), yellow (stripe) rust (*Yr*), stem rust (*Sr*), powdery mildew (*Pm*) and even some hemi-biotrophic fungi.

Besides these rare monogenic examples, also MDR for QDR have been detected. Two out of 110 CIMMYT lines were highly resistant to five wheat diseases, including FHB [106]. MDR loci for resistance to tan spot (caused by *Pyrenophora tritici-repentis*) and SNB have been found in 88 out of a panel of 825 wheat accessions from the USDA [107]. However, in both studies, no molecular analyses of the genes/QTLs underlying MDR have been performed. In a GWAS, ten of the 35 detected QTLs conferred resistance to each of two diseases (leaf rust, stem rust, and yellow rust, yellow leaf spot, STB, crown rot) [108]. In an independent GWAS of 158 winter wheat accessions, about 10% of the cultivars had superior resistance to yellow rust, stem rust, powdery mildew, and FHB simultaneously [109]. Nine QTLs explaining 62% of the total genotypic variation were detected for MDR. Only three of them were also found as QTLs for a single disease resistance. This might be a hint on genomic regions caused by “real” MDR and not only by pyramiding independent loci that are triggered by the strong selection of the breeders for combined resistances. In another study, among 125 synthetic hexaploid wheats a wide range of genetic variation was observed for two to five biotic stresses with 17 lines being resistant to more than one disease [110]. The corresponding GWAS detected 124 significant marker-trait associations for multiple biotic stresses and 33 of these were found within known genes.

Several meta-analyses have suggested that MDR loci are relatively common in maize [111,112]. The latter study demonstrated that many QTLs are not randomly distributed over the maize genome, but clustered in specific regions. More recently, this trend was confirmed by organizing the distribution of 1080 QTLs for disease resistances mapped in 110 studies [113]. Chromosome 1 and 3 were revealed carrying the highest proportion of QTLs for resistance. Currently, a multiple diseases approach instead of analyzing single disease resistances is increasingly getting attention. Different authors combined the localization of single-disease QTLs finding candidate regions for MDR on the following bins: 1.02, 1.05/1.06, 3.04, 4.06, 7.02, 8.03, 8.05 and 9.02 [29,90,100,113,114,115,116,117]. Some of these regions overlap with hotspots for resistance to NCLB presented in Figure 2. Obviously, some mechanisms underlying QDR are unspecific and common for several diseases. Some regions also presented contrasting effects for resistances to bacterial and fungal diseases [115].

The maize chromosome bin 1.02 has been identified as a region that confers resistance to a number of maize diseases such as GER [83], ear and stalk rot (caused by multiple pathogens), common smut (caused by *Ustilago maydis*), gray leaf spot (GLS, caused by *Cercospora zeae-maydis*) [118,119], southern corn leaf blight (SCLB, caused by *Bipolaris maydis*, syn. *Helminthosporium maydis*), NCLB, Stewart’s wilt (caused by *Pantoea stewartii*) and common rust (caused by *Puccinia sorghi*) [102,112,120]. The latter study investigated candidate genes at this region enhancing the accumulation of callose and phenolic components around infection sites [120], an unspecific defense mechanism that might be helpful against all of these pathogens. More recently, 37 QTLs were identified for NCLB, SCLB, and GLS resistances in two maize populations, four QTLs overlapped for each of two diseases [121]. 

Another hotspot seems to be maize bins 1.05/1.06, where resistance to NCLB, Stewart’s wilt, SCLB, common rust, GLS, and ear and stalk rot caused by multiple fungi have been detected [90,100,112,114,115,120]. The bin 1.06 was reported by several authors for NCLB resistance (rf. to Figure 2) and the dominant Stewart’s wilt resistance gene *Sw1* had been mapped in the same genomic region [90]. A subsequent fine mapping of this chromosomal region resulted in overlapping regions for both diseases. The association study revealed the candidate genes *pan1* that might be a susceptibility gene for NCLB and Stewart’s wilt underlying this QTL [90]. Additionally, copy number variation was found as a structural element of this genomic region.

Also, bin 8.05–8.06 harbors QTL and genes for resistance to many diseases, among them NCLB, SCLB, GLS, common rust, common smut, maize streak virus, and aflatoxin accumulation [122]. Several resistance gene analogs and defense response gene homologs were identified in this region as well as the *Ht2* gene for NCLB resistance. 

A few other MDR loci have been assigned to candidate genes, among them a glutathione *S*-transferase (*GST*) gene for resistance to SCLB, NCLB, and GLS [116]. More recently, a QTL in bin 9.02 was associated by genomic techniques with a caffeoyl-CoA *O*-methyltransferase (*ZmCCoAOMT2),* a gene conferring QDR to both SCLB and GLS being in connection with the phenylpropanoid pathway and lignin production [117]. This is another example for a MDR gene that might encode an unspecific disease resistance mechanism. 

In conclusion, we are just at the starting point of understanding the mechanisms of MDR. Some MDR QTLs split into many QTLs/genes when fine mapped, others are associated with genes conferring unspecific resistance reactions and in some cases resistance genes are present in varying copy numbers. 

## 6. Integration of Genomic Data in the Ongoing Breeding Process

The art of breeding is to integrate new genomic-based methods into existing breeding schemes to speed up the process and make it more efficient. Here, we will discuss two important aspects: Genomics-based introgression of genetic resources and genetic improvement within elite populations.

### 6.1. Introgression of Genetic Resources

Genetic resources are a valuable tool to expand genetic variation in highly selected elite populations, especially for resistance breeding. The occurrence of new diseases or new races of a well-known pathogen often makes it necessary to find new resistance sources among non-adapted materials. Possible sources are old landraces or foreign materials that are less or even not adapted to the target region. In either case, adaptation breeding is essential with highly heritable traits, like flowering date, plant height, lodging tolerance etc., selected first.

In hybrid breeding, it must be additionally admitted that DH lines derived from non-adapted, non-inbred materials suffer from a remnant genetic load that leads to highly undesirable agronomic phenotypes such as low emergence rate, poor growth rate, lodging, poor seed set, and low grain yield [84,123]. Inbreeding depression among DH lines can also result in unwanted traits such as high leaf chlorosis, tillering, extreme susceptibility to diseases such as ear rots, common smut (*Ustilago maydis*) and common rust (*Puccinia sorghi*) [123]. Using materials from foreign regions may additionally afford adaptation to day length and agronomic practices.

Therefore, introgression of resistance alleles from genetic resources into elite materials should target resistant lines having better agronomic properties, to reduce the effect of detrimental alleles. Using European flint landraces as a source, led to the exclusion of close to 70% of the produced DH lines because of their high inbreeding depression [84].

**Figure 3:**
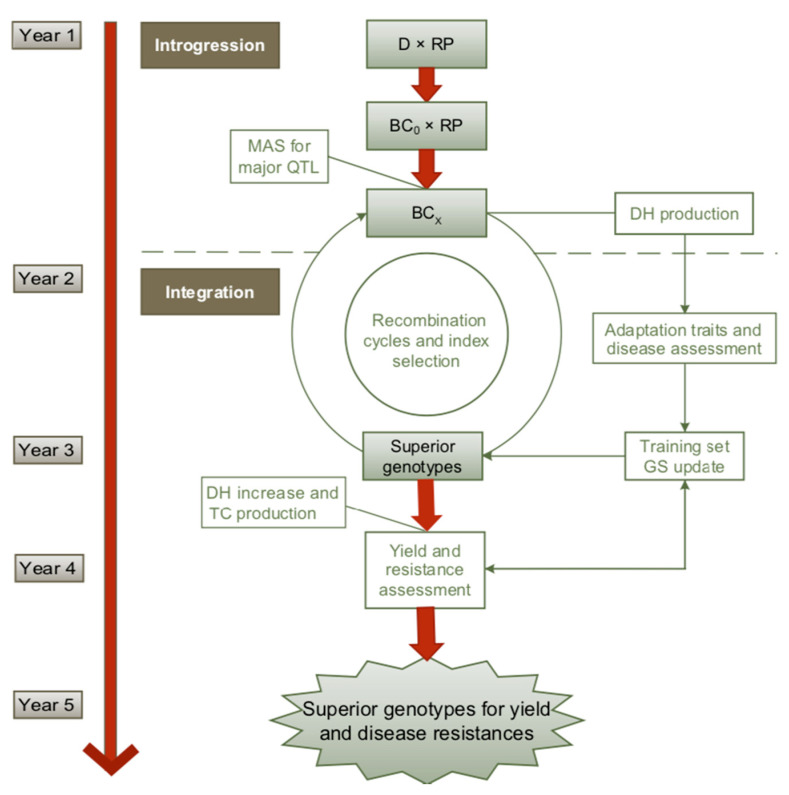
Schematic illustration of a rapid genomics-assisted breeding approach for using genetic resources in maize [124]. D = donor of resistance, RP = recurrent parent, BC =, backcross, MAS = marker assisted selection, DH = doubled haploid lines, GS = genomic selection, TC = testcross.

In an integrated scheme with maize as an example (Figure 3), firstly, the resistance donor is introgressed by backcrossing to the recurrent parent (RP) and by selection of the major QTLs by MAS. When only small-effect QTLs are available the introgression part can be confined to backcrossing without marker selection. 

In the integration step, GS is performed in a recurrent selection scheme to rapidly select for adaptation traits and disease resistances. The training population can be developed in parallel to a first phenotypic selection cycle or by using historical data from the same resistance sources. To establish or update the training set, a large-scaled DH production and intensive phenotyping for adaptation traits and resistance to the targeted diseases are necessary. The outcome of the training set is then the basis for genomic prediction of superior DH lines. They are multiplied and crossed to testers to select superior hybrids for yield and other complex traits on testcross basis. The timeline refers to the selection on BC_1_ and two generations per year. The cycle might be longer if more BC generations are necessary when afforded by a low adaptation of the genetic resources to the target environments.

### 6.2. Improvement within Elite Materials

**Figure 4:**
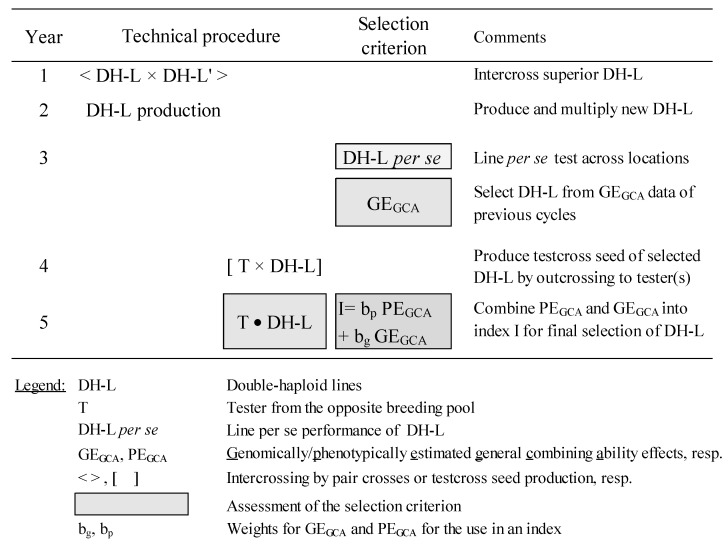
Breeding scheme for combined genomic and phenotypic selection in hybrid breeding (Peer Wilde, pers. commun.).

Genomics-based improvement of existing breeding schemes is now revolutionizing the practical breeding work. An example is shown in Figure 4 for so called ‘second-cycle’ breeding in hybrid development. 

The scheme starts with intercrossing the best double-haploid lines (DH-L) and producing new DH-L from the F1s. Selection of line per se performance in several locations concentrates on high heritable traits, like vitality, rust resistances, resistance to lodging, and grain quality. Phenotypic selection can be supported already on this stage by selecting on general combining ability (GCA) for grain yield with genomically estimated effects (GE_GCA_) from previous cycles. Assuming a sufficiently high prediction accuracy, lines with an inferior GE_GCA_ can be already discarded saving the efforts for producing and phenotyping testcrosses to the putatively better part of the line population. The best lines are outcrossed to tester(s) of the opposite heterotic group and phenotyped in multi-location trials assessing GCA estimates for grain yield and complex disease resistances. These phenotypically estimated effects (PE_GCA_) can be supplemented with GE_GCA_ effects from genomic prediction to enter a weighted selection index combining both sources of information. The integration of genomic selection will be especially beneficial if the model training comes from aggregated data across multiple breeding cycles [125]. This would also mitigate biases from genotype × year interaction. DH-L with a positive index value will continue to a second selection stage and will be intercrossed for setting up a new breeding population.

Model studies in rye showed that the gain from selection for grain yield with S_2_ lines is in a combined scheme up to 12% higher than with pure phenotypic selection assuming a prediction accuracy of 0.5 [Peer Wilde, pers. commun.]. These estimates for grain yield should be even higher for QDR because these usually display a higher heritability. GS would then increase the gain from selection due to a reduced number of years per cycle and/or by genotyping more lines with the same costs allowing a greater selection intensity.

## 7. Conclusions

Genomics-based breeding methods are a great step forward without relying on gene technology procedures. They can be introduced into practical breeding schemes right away. By using QDR, the way is longer and more tedious and might be hindered by additional trade-offs, however, the durability over time should also be higher. Recent advantages in genomics illustrate the high complexity of quantitative host-pathogen interactions. However, our growing knowledge on the most important pathosystems also allows the development of novel breeding strategies, where genomic selection seems to be highly advantageous for saving time and field space in many, but not all, pathosystems.

The benefit of genomic selection greatly relies on the genetic relationship between training and validation population. Therefore, the training population must be updated subsequently. Here, resistance breeding has an unique advantage over other agronomic traits because distinctive resistance sources are used in breeding. Once they are identified and validated, they could be used over and over again and, therefore, the respective genomic models can also be used across cycles with much less loss of prediction accuracy than with, for example, grain yield. And because medium-dense marker assays are anyway used for prediction of grain yield, the only additional cost for resistance selection is the multi-environmental analysis of the training population for the respective disease resistances. The identification of multiple-disease resistance genes might be further helpful in this respect.

The high impact in genomic research might in future result in the knowledge of more genes underlying disease-resistance QTLs with a high effect. This opens the avenue to genome editing techniques that are, however, highly challenging for manipulating whole gene networks as it is necessary for the improvement of QDR.

## Figures and Tables

**Table 1:** Main advantages and pitfalls^1^ for GS in science and breeding.

Advantages	Pitfalls ^1^
No special population structure or segregating generations necessary	Same environments for testing training and validation sets
Usage of the full genome for trait association including small QTLs that might not surpass the significance level in mapping	Creating training and validation set from the same base population or having a high degree of relatedness between both sets
GS data can directly be used for selection without phenotyping	Inclusion of non-related populations can cause severe changes in allele frequencies

^1^ leading to an incorrect estimation of prediction accuracies (over-/underestimation).

## Supplementary Materials

Supplementary Materials can be found at https://www.mdpi.com/1422-0067/21/24/9717/s1.

## Abbreviations

FHB	*Fusarium *head blight
STB	Septoria tritici blotch
SNB	Septoria nodorum blotch
GER	Gibberella ear rot
FER	*Fusarium* ear rot
NCLB	Northern corn leaf blight
QTL	Quantitative trait locus
MDR	Multi-disease resistance
*R*	Resistance gene (race-specific)
QDR	Quantitative disease resistance
ETI	Effector-triggered immunity
GS	Genomic selection
GP	Genomic prediction
MAS	Marker-assisted selection
MARS	Marker-assisted recurrent selection
GWAS	Genome-wide association study
SNP	Single-nucleotide polymorphism
LD	Linkage disequilibrium
GEBV	Genomic estimated breeding values
